# Priorities in testis cancer care during Covid-19 Pandemic

**DOI:** 10.1590/S1677-5538.IBJU.2020.S109

**Published:** 2020-07-27

**Authors:** Fernando P. Secin

**Affiliations:** 1 University of Buenos Aires School of Medicine Discipline of Urology Buenos Aires Argentina Discipline of Urology, University of Buenos Aires School of Medicine, Buenos Aires, Argentina

**Keywords:** Testicular Neoplasms, Patients, COVID-19 [Supplementary Concept]

## Abstract

**Introduction:**

There is little information on how to prioritize testis cancer (TC) patients' care during COVID-19 pandemic in order to relieve its pressure on the health care systems.

**Objective:**

To describe the recommendations for diagnosis, treatment and follow-up of patients with TC amidst COVID- 19 pandemic.

**Material and Methods:**

Pubmed search and review of the main urological association guidelines on TC.

**Results:**

The biology of TC requires immediate care of patients during diagnosis, initial surgical therapy and management of recurrent disease. Active surveillance is the first choice of management and should be offered to all compliant clinical stage I TC patients provided they understand the need to self-isolate. Active surveillance may also help decrease the demand for intensive care unit beds, ventilators, personal protective equipment, and other critical hospital and human resources by minimizing surgeries without compromising patient outcomes. Complications of therapy and symptomatic patients represent medical emergencies and should be treated immediately. Telemedicine may be useful during follow-up periods.

**Conclusions:**

Most stages of testis cancer require urgent care; however, all recommendations must be adapted to local health care priorities considering that most of these patients are at low risk of severe COVID-19 infection.

## INTRODUCTION

Testicular cancer (TC), although rare, is the commonest, solid-organ cancer in men aged 15 -44 years. As the tumor is promptly identified and treated, the overall prognosis is excellent even after late diagnosis. While some authors have reported a significant relation between survival and delay in diagnosis, ( 1 , 2 ) others have shown no impact on survival ( 3 ).

Actually, the American Urological Association (AUA), ( 4 ) European Association of Urology (EAU) ( 5 ) and National Comprehensive Cancer Network (NCCN) ( 6 ) guidelines on TC do not make specific considerations in terms of prompt treatment or impact of treatment delay on outcome. Only, the EAU TC guidelines speak about “adequate early treatment” without defining the term “early” ( 5 ).

Notwithstanding, it is generally assumed that delays in diagnosis affect the stage of disease at presentation and therefore disease prognosis ( 7 ). That is why, all patients suspected of having TC are recommended to be seen urgently (within 2 weeks) by a specialist ( 8 ).

The coronavirus disease 2019 (COVID-19) pandemic has created major dilemmas for providers in all areas of health care delivery, including cancer centers, forcing them to make substantial changes. While medical institutions may request that elective surgeries be postponed until the strain on the health care system from COVID-19 has been relieved, the characteristics of elective surgeries in urology oncology are context-dependent and have not been well defined in the current crisis.

Fortunately, TC patients are usually young and healthy. Their risk of severe disease compared favorably with the risk reported in the general population of patients presenting with Covid-19. However, they cannot be excluded from the unprecedented measures taken in health systems worldwide ( 9 ).

We herein describe the recommendations for diagnosis, treatment and follow-up of TC patients amidst Covid 19 pandemic based on published studies as well as expert opinion delivered through main urological societies. Needless to say, sound clinical judgment and final decisions should be tailored to the local infection severity and pandemic phase ( 10 ).

## MATERIAL AND METHODS

We did a Pubmed word search using the terms: “TC and pandemia”, TC and covid”, “TC and coronavirus” and “urologic surgery and pandemia”, and reviewed main urological society guidelines and recommendations in terms of surgical priorities during the COVID-19 pandemic.

Seventy-four manuscripts were retrieved and recommendations from the European Association of Urology and the British Association of Urological surgeons were reviewed.

To facilitate understanding, TC will be approached according to disease stage:

1. Diagnosis
2. TC initial treatment:
3. Management of clinical Stage I (CSI) TC
4. Management of primary metastatic TC
5. Management of residual disease post chemotherapy.

### Follow-up

Unfortunately, there might not be high--quality evidence for the compromises proposed but it is anticipated the new information will function as an additional guide to the management of urological conditions during the current COVID-19 pandemic.

## RESULTS

The EAU categorized recommendations with increasing degrees of priorities as follows ( 11 ):

- LOW PRIORITY: Clinical harm (progression, metastasis, loss of function) very unlikely if postponed 6 months.
- INTERMEDIATE PRIORITY: Cancel but reconsider in case of increase in capacity (not recommended postponing more than 3 months: Clinical harm (progression, metastasis, loss of organ function) possible if postponed 3-4 months but unlikely.
- HIGH PRIORITY: The last to cancel, prevent delay of > 6 weeks. Clinical harm (progression, metastasis, loss of organ function and deaths) very likely if postponed > 6 weeks.
- EMERGENCY: Cannot be postponed for more 24 hours. Life-threatening situation.

### TC diagnosis:

All patients with suspicion of TC should undergo a bilateral testicular ultrasound within 24 hours of clinical examination, which should include physical examination of supraclavicular, cervical, axillary and inguinal lymph nodes, breasts and testicles.

Like normally done outside any pandemic state, serum tumor markers should be evaluated before and after orchiectomy.

Non contrast-enhanced CT scan of the chest and contrast-enhanced CT scan of the abdomen and pelvis should be done in patients with a diagnosis of TC ideally before orchidectomy. In case of iodine allergy or other limiting factors perform MRI of the abdomen and pelvis. According to the EAU recommendations, if diagnostic imaging studies had not been performed before orchidectomy, they may be postponed awaiting pathology result but no more than 7 days.

### TC initial treatment:

Radical orchiectomy should be performed as soon as possible because it is an outpatient procedure and will guide further treatment ( 12 ). EAU experts consider orchidectomy a surgical emergency, however, it may be postponed 2-3 days, as well as the pathological examination of the testis. Histologic evaluation time may vary significantly from institution to institution and health care systems (private, public, etc.).

MRI of the brain (or brain CT if not available) should be indicated on an emergency basis in patients with central nervous system symptoms, multiple lung metastases, high β-hCG values, or those in the poor-prognosis IGCCCG risk group. MRI of the brain could eventually be postponed until chest CT or marker results are available, but then becomes an emergency.

Patients at high-risk for contralateral germ cell neoplasia in situ are recommended to undergo biopsy of the contralateral testis during orchidectomy. They include patients with an atrophic contralateral testis (< 20 cc volume on ultrasound) who present before the age of 31 years, ( 13 ) azoospermic ( 14 ) and present ultra-sonographic abnormalities. ( 15 , 16 ) If contralateral biopsy was not done during contralateral orchiectomy, it can be postponed 6 months.

Sperm banking is another low priority procedure, particularly in those patients who had not done it prior to orchiectomy and do not need adjuvant chemotherapy or radiotherapy. In patients scheduled for adjuvant treatment sperm banking becomes an emergency and should be done prior to starting treatment.

There is currently no evidence of vertical transmission of COVID-19. However, patients may be offered testing at their discretion at the time of performing standard serology (i.e. HIV/Hepatitis testing) prior to sperm cryopreservation if specific covid-19 diagnostic tests are available.

### Management of clinical Stage I (CSI) TC

Active surveillance is the first choice of management in compliant CSI TC patients, particularly during COVID-19 pandemic. Active surveillance should be offered to all these patients with seminoma and low risk (no lymphovascular invasion) non seminoma germ cell tumors (NSGCT) provided they understand the need to self-isolate ( 10 ).

Unless the patient has contraindications to other forms of therapy, RPLND should be discouraged in order to eventually avoid use of an intensive care unit bed or a ventilator machine, and decrease patient length of hospital stay, thus, decreasing his chances of becoming Covid-19 infected.

CSI seminoma or NSGCT patients not accepting active surveillance need to be treated. They are considered high priority and should be treated within 6 weeks of histologic confirmation (High priority). This group of patients with CSI seminoma should be treated with one course of carboplatin at AUC7. Experts agree that in spite of the lack of evidence on the association of bleomycin with severe lung COVID disease, bleomycin should be avoided when possible and hematopoietic growth factors (G-CSF) should be co-administered to diminish the incidence of neutropenia and infection in all patients with germ cell tumor (GCT) receiving chemotherapy.

Patients with low-risk NSGCT CSI not willing or unsuitable to undergo active surveillance should receive one cycle of BEP and G-CSF. Patients with high risk CSI-NSCGT (presence of lymphovascular invasion) should be treated with one course of BEP and G-CSF if they are not willing to accept AS.

Primary nerve-sparing RPLND should only be indicated in CSI -NSGCT patients with contraindication to adjuvant chemotherapy and unwilling to accept active surveillance, or in those with teratoma with somatic-type malignancy.

### Management of primary metastatic TC

Except for patients with clinical stage IIA seminoma who can be treated with either radio-therapy or chemotherapy within 6 weeks of histologic confirmation, all other patients with metastatic disease at presentation should be treated immediately.

Patients in a good general condition may delay the initiation of chemotherapy for 7 days. In addition, short planned delays in chemotherapy for good-risk GCT patients (≤7 days per cycle) also appear to be acceptable since they may prevent serious toxicity in this curable patient population ( 17 ).

Clinical stage IIB seminoma patients should be treated with chemotherapy according to the International Germ Cell Consensus Classification (IGCCC) good risk group. (3x BEP o 4x EP + G-CSF) ( 18 , 19 ) Radiotherapy may be considered as alternative in selected clinical stage IIB seminoma depending on availability.

Patients with stage ≤ IIC seminoma should receive primary chemotherapy based on the same principles used for NSGCT. IGCCC good risk NSGCT should be treated with 3x BEP o 4x EP + G-CSF while the recommended therapy for IGCCC intermediate or poor risk groups is 4x VIP or 4x BEP + G-CSF. ( 6 ) Carneiro et al. recommend the use of VIP (Etoposide; Ifosfamide and Cisplatin) in patients with intermediate or poor risk metastatic GCT, instead of the 4x BEP, to avoid the use of bleomycin ( 12 ). Again, patients in a good general condition may delay the initiation of treatment for 7 days.

In a life-threatening situation due to extensive metastasis, patients should be hospitalized and commence chemotherapy prior to orchidectomy (clinical principle).

### Management of residual disease post chemotherapy

Post-chemotherapy full bilateral RPLND of either residual masses after chemotherapy for NSGCT with negative serum levels of tumor markers or growing teratoma are considered high priority and the surgery should be performed within 6 weeks of completed chemotherapy.

### Follow-up of TC

Patients with Seminoma and NSGCT CSI on AS or after adjuvant chemotherapy are recommended to be followed within 6 weeks of the original appointment (High priority). EAU experts recommend not to postpone follow-up beyond 3 to 6 months of the original appointment in patients with metastatic disease after adjuvant treatment or complete remission.

Direct-to-consumer (or on-demand) telemedicine may allow patients to be efficiently followed, as it is both patient-centered and conducive to self-quarantine, and it protects patients, clinicians, and the community from exposure. It can allow physicians and patients to communicate any time as needed, using smartphones or webcam-enabled computers ( 20 ).

Patients with symptomatic brain metastases following treatment, post-obstructive polyuria or symptomatic postoperative complications (infection, bleeding, lymphoceles/lymphatic ascitis, etc.), intractable pain or symptomatic neutropenia during or after chemotherapy (fever, sepsis) represent medical emergencies and should be treated immediately.

## DISCUSSION

Urologists can make a substantial contribution to the health care systems by decreasing the demand for hospital beds, ventilators, personal protective equipment, and other critical hospital and human resources by minimizing surgeries without compromising patient outcomes whenever possible ( 21 ).

Medical specialists in general and urologists in particular are encouraged to weigh the impact of nonsurgical therapies such as systemic chemotherapy (that can leave patients at greater risk of contracting and potentially succumbing to COVID-19) and surgical risks against the natural history of the disease in case it is not timely treated.

Generally speaking, considerations should include nonsurgical treatments whenever available or deferral of surgery until patient risks of in-hospital COVID-19 infection, demand for ventilators and inpatient beds diminish. The recommendation for different stages of disease in patients with testis cancer are summarizes in the Table-1 .

**Table 1:** Summary of recommendation for different stages of disease in patients with testis cancer.

**1. Diagnosis:** – Low priority: Sperm banking – Emergency: Ultrasound, physical exam and serum tumor markers
**2. Initial treatment:** – Low priority: Contralateral biopsy (see text) – **Emergency:** Orchiectomy with 2 -3 days Imaging within 7 days in asymptomatic patients
**3- Stage 1 management:** – Low priority: In case AS is offered according to guidelines – High priority: In case treatment is offered according to guidelines
**4- Treatment metastatic disease:** – **High priority:** Any adjuvant treatment in stage IIA Seminoma (Radio or chemotherapy) Adjuvant treatment in stage IIA/B NSGCT with negative markers – **Emergency:** Treatment in stage > IIB Seminoma and NSGCT within 7 days (Chemotherapy) Symptomatic, life threatening or poor risk
**5- Management of residual disease post chemotherapy** – Low to high priority: Can be delayed from 6 weeks to 6 months depending case by case (telemedicine) – **Emergency:** Symptomatic postoperative complications, pain, neutropenia with fever o sepsis, etc.

The impact of surgical wait time on the outcome of testis tumors remains controversial. ( 22 – 24 ) There are few studies evaluating components of wait times (e.g. delay in diagnosis, delay in orchiectomy) in TC patients; however, differences in study data availability, method of analysis and wait time definitions precluded statistical pooling of the findings ( 23 ). Nonetheless, given the unpredictable biology and speed of TC cell dissemination, both diagnostic and treatment delays are strongly discouraged ( 17 ).

Regarding treatment options in patients with low volume stage II seminomas (IIA and IIB), some authors recommend radiotherapy to avoid the use of chemotherapy ( 12 ). However, several radiotherapy centers are currently closed due to the COVID-19 pandemic.

The same authors also recommend 4 cycles of VIP (Etoposide 75mg/m2 IV on Days 1-5; Ifosfamide 1200mg/m2 IV on Days 1-5 with same protection and Cisplatin 20mg/m2 IV on Days 1-5, every 21 days) in patients with intermediate or poor risk metastatic GCT, instead of the 4x BEP, to avoid the use of bleomycin. Although there is no evidence on the association of bleomycin with severe lung COVID disease, it is generally agreed to avoid bleomycin when possible. Hematopoietic growth colony stimulating factors (G-CSF) are recommended in all patients with germ cell tumor (GCT) receiving chemotherapy to diminish the incidence of neutropenia and infection ( 11 , 12 ).

Disasters and pandemics pose unique challenges to health care delivery and the multiple potential benefits of telemedicine are not new ( 25 ). More than 50 U.S. health systems already have telehealth programs to allow clinicians to see patients who are at home. However, it is not widely utilized in other parts of the World. Telemedicine visits can be conducted with both patient and clinician at home, greatly limiting travel and exposure and permitting uninterrupted care of patients ( 20 ). Telemedicine is an attractive strategy to follow CSI patients on active surveillance as long as they can periodically provide imaging studies and serum tumor markers results. Some of telemedicine limitations include cost, training, reimbursement, credentialing and impossibility to perform physical exam, which is extremely important during active surveillance.

Needless to say, all recommendations are mindful of significant differences between countries and regions. Depending on resources, doctors will need to make decisions according to local health care priorities. Countries or even provinces that have not had high rates of death from COVID-19 could consider similar approaches that involve balancing pandemic control with providing continued cancer care ( 26 ).

## References

[1] 1. Thornhill JA, Fennelly JJ, Kelly DG, Walsh A, Fitzpatrick JM. Patients’ delay in the presentation of testis cancer in Ireland. Br J Urol. 1987; 59:447-51. 10.1111/j.1464-410x.1987.tb04844.x 3594102

[2] 2. Vasudev NS, Joffe JK, Cooke C, Richards F, Jones WG. Delay in the diagnosis of testicular tumours -changes over the past 18 years. Br J Gen Pract. 2004; 54:595-7. PMC1324839 15296558

[3] 3. Fossa SD, Klepp O, Elgjo RF, Eliassen G, Melsom H, Urnes T, et al. The effect of patient's delay and doctor's delay in patients with malignant germ cell tumours. Int J Androl. 1981; 4 Suppl s4:134-44. 10.1111/j.1365-2605.1981.tb00664.x 29112237

[4] 4. Stephenson A, Eggener SE, Bass EB, Chelnick DM, Daneshmand S, Feldman D, et al. Diagnosis and Treatment of Early Stage Testicular Cancer: AUA Guideline (2019). [Internet]. Available at. <https://www.auanet.org/guidelines/testicular-cancer-guideline>. (Accessed May 2, 2020) 10.1097/JU.0000000000000318 31059667

[5] 5. Laguna MP, Albers P, Algaba F, Bokemeyer C, Boormans JL, Fischer S, et al. Testicular Cancer. European Association of Urology. [Internet]. Available at <https://uroweb.org/guideline/testicular-cancer/>.

[6] 6. [No Authors]. National Comprehensive Cancer Network. [Internet]. Available at. <www.nccn.org/professionals/physician_gls/pdf/testicular.pdf>. (Accessed May 2, 2020)

[7] 7. [No Authors]. Prognostic factors in advanced nonseminomatous germ-cell testicular tumours: results of a multicentre study. Report from the Medical Research Council Working Party on Testicular Tumours. Lancet. 1985; 1:8-11. 2578205

[8] 8. Dearnaley D, Huddart R, Horwich A. Regular review: Managing testicular cancer. BMJ. 2001; 322:1583-8. 10.1136/bmj.322.7302.1583 PMC1120626 11431302

[9] 9. Guan WJ, Ni ZY, Hu Y, Liang WH, Ou CQ, He JX, et al. Medical Treatment Expert Group for Covid-19. Clinical Characteristics of Coronavirus Disease 2019 in China. N Engl J Med. 2020; 382:1708-20. 10.1056/NEJMoa2002032 PMC7092819 32109013

[10] 10. Ward C. COVID-19 Strategy for the Interim Management of Testicular Cancer Prepared by the BAUS Section of Oncology. [Internet]. Available at. <https://www.bopa.org.uk/resources/covid-19-strategy-for-the-interimmanagement-of-testicular-cancer-prepared-by-the-baussection-of-oncology/>.

[11] 11. [No Authors]. Recommendations from the EAU NMIBC Guidelines Panel applicable during the COVID-19 pandemic. [Internet]. Available at. <uroweb.org/wp-content/uploads/Combined-oncology-COVID-19recommendations.pdf>.

[12] 12. Carneiro A, Wroclawski ML, Nahar B, Soares A, Cardoso AP, Kim NJ, et al. Impact of the COVID-19 Pandemic on the Urologist's clinical practice in Brazil: a management guideline proposal for low- and middle-income countries during the crisis period. Int Braz J Urol. 2020; 46:501-10. 10.1590/S1677-5538.IBJU.2020.04.03 PMC7239291 32271512

[13] 13. Harland SJ, Cook PA, Fossa SD, Horwich A, Mead GM, Parkinson MC, et al. Intratubular germ cell neoplasia of the contralateral testis in testicular cancer: defining a high risk group. J Urol. 1998; 160:1353-7. 9751353

[14] 14. Rud CN, Daugaard G, Rajpert-De Meyts E, Skakkebæk NE, Petersen JH, Jørgensen N. Sperm concentration, testicular volume and age predict risk of carcinoma in situ in contralateral testis of men with testicular germ cell cancer. J Urol. 2013; 190:2074-80. 10.1016/j.juro.2013.06.023 23770148

[15] 15. Tan IB, Ang KK, Ching BC, Mohan C, Toh CK, Tan MH. Testicular microlithiasis predicts concurrent testicular germ cell tumors and intratubular germ cell neoplasia of unclassified type in adults: a meta-analysis and systematic review. Cancer. 2010; 116:4520-32. 10.1002/cncr.25231 20578177

[16] 16. Elzinga-Tinke JE, Dohle GR, Looijenga LH. Etiology and early pathogenesis of malignant testicular germ cell tumors: towards possibilities for preinvasive diagnosis. Asian J Androl. 2015; 17:381-93. 10.4103/1008-682X.148079 PMC4430936 25791729

[17] 17. Motzer RJ, Geller NL, Bosl GJ. The effect of a 7-day delay in chemotherapy cycles on complete response and event-free survival in good-risk disseminated germ cell tumor patients. Cancer. 1990; 66:857-61. 10.1002/1097-0142(19900901)66:5<857::aid-cncr2820660508>3.0.co;2-g 1696846

[18] 18. [No Authors]. International Germ Cell Consensus Classification: a prognostic factor-based staging system for metastatic germ cell cancers. International Germ Cell Cancer Collaborative Group. J Clin Oncol. 1997; 15:594-603. 10.1200/JCO.1997.15.2.594 9053482

[19] 19. Culine S, Kerbrat P, Kramar A, Théodore C, Chevreau C, Geoffrois L, et al. Genito-Urinary Group of the French Federation of Cancer Center (GETUG T93BP). Refining the optimal chemotherapy regimen for good-risk metastatic nonseminomatous germ-cell tumors: a randomized trial of the Genito-Urinary Group of the French Federation of Cancer Centers (GETUG T93BP). Ann Oncol. 2007; 18:917-24. 10.1093/annonc/mdm062 17351252

[20] 20. Hollander JE, Carr BG. Virtually Perfect? Telemedicine for Covid-19. N Engl J Med. 2020; 382:1679-81. 10.1056/NEJMp2003539 32160451

[21] 21. Stensland KD, Morgan TM, Moinzadeh A, Lee CT, Briganti A, Catto JWF, et al. Considerations in the Triage of Urologic Surgeries During the COVID-19 Pandemic. Eur Urol. 2020; 77:663-6. 10.1016/j.eururo.2020.03.027 PMC7146681 32279903

[22] 22. Harding M, Paul J, Kaye SB. Does delayed diagnosis or scrotal incision affect outcome for men with nonseminomatous germ cell tumours? Br J Urol. 1995; 76:491-4. 10.1111/j.1464-410x.1995.tb07754.x 7551890

[23] 23. Bell D, Morash C, Dranitsaris G, Izawa J, Short T, Klotz LH, et al. Canadian surgical wait times (SWAT) initiative. Does prolonging the time to testicular cancer surgery impact long-term cancer control: a systematic review of the literature. Can J Urol. 2006; 13 Suppl 3:30-6. 16818010

[24] 24. Derrett S, Paul C, Morris JM. Waiting for elective surgery: effects on health-related quality of life. Int J Qual Health Care. 1999; 11:47-57. 10.1093/intqhc/11.1.47 10411289

[25] 25. Lurie N, Carr BG. The Role of Telehealth in the Medical Response to Disasters. JAMA Intern Med. 2018; 178:745-6. 10.1001/jamainternmed.2018.1314 29710200

[26] 26. Pramesh CS, Badwe RA. Cancer Management in India during Covid-19. N Engl J Med. 2020; 382:e61. 10.1056/NEJMc2011595 PMC7207224 32343498

